# Unilateral orbital surgical emphysema following laparoscopic hiatal hernia repair and Nissen fundoplication: a case report

**DOI:** 10.3389/fsurg.2026.1741472

**Published:** 2026-03-05

**Authors:** Ahmed M. Saggaf, Hassan U. Al-ghamdi, Naseer Ayed Asiri, Mihdhar O. Saggaf, Ali Nagi, Raneem Alathath, Jumana H. Timraz, Husna Irfan Thalib

**Affiliations:** 1 General Surgery Department, King Abdulaziz Hospital, Jeddah, Saudi Arabia; 2 Department of Thoracic Surgery, King Fahad Hospital, Al Baha, Saudi Arabia; 3 Prince Meshari Bin Saud, General Hospital, Al Baha, Saudi Arabia; 4 Ibn Sina College, Jeddah, Saudi Arabia; 5 General Medicine Practice Program, Batterjee Medical College, Jeddah, Saudi Arabia

**Keywords:** laparoscopic surgery, Nissen fundoplication, orbital emphysema, postoperative complication, subcutaneous emphysema

## Abstract

Subcutaneous emphysema (SE) is a recognized but rare complication of laparoscopic surgery, and orbital involvement is particularly unusual. We report a rare case of unilateral orbital SE following laparoscopic hiatal hernia repair with Nissen fundoplication. A 49-year-old hypertensive woman with gastroesophageal reflux disease (GERD) underwent elective laparoscopic hiatal hernia repair. Intraoperatively, she developed sudden-onset left orbital emphysema. Imaging revealed mild left pneumothorax and bilateral pleural effusions. Orbital swelling was identified intraoperatively, prompting multidisciplinary evaluation including anesthesiology, surgery, and ophthalmology. Conservative management resulted in rapid and complete resolution within 48 h, without visual or respiratory sequelae. Orbital SE following laparoscopy is uncommon but clinically significant. Risk factors include high insufflation pressure, previous abdominal surgeries, and prolonged operative time. Prompt clinical and radiological assessment ensures early diagnosis and prevents complications such as orbital compartment syndrome. Surgeons must remain vigilant for rare SE presentations after laparoscopy. Conservative management is effective in mild cases, but early multidisciplinary intervention is essential to prevent vision loss.

## 1 Introduction

Subcutaneous emphysema (SE) refers to the presence of air or gas within the subcutaneous tissues. Commonly presenting with facial or neck swelling and crepitus, it may also involve more unusual sites such as the orbit. Orbital emphysema is particularly dangerous due to its potential to raise intraorbital pressure and compromise ocular perfusion, possibly leading to orbital compartment syndrome and vision loss (1, 2).

Although antireflux surgery, such as Nissen fundoplication, is considered safe and effective in managing gastroesophageal reflux disease (GERD), complications like SE can occur, particularly with laparoscopic approaches. The escape of insufflated carbon dioxide (CO₂) gas may track through weak fascial planes, congenital diaphragmatic defects, or trocar entry sites (3, 4). We describe a rare intraoperative case of unilateral orbital SE following laparoscopic hiatal hernia repair with Nissen fundoplication in a middle-aged female patient. The novelty of this case lies in the intraoperative onset of strictly unilateral orbital emphysema, its association with concurrent pneumothorax and pleural effusions, and its rapid resolution with conservative management without visual compromise.

## 2 Case report

**Figure 1:**
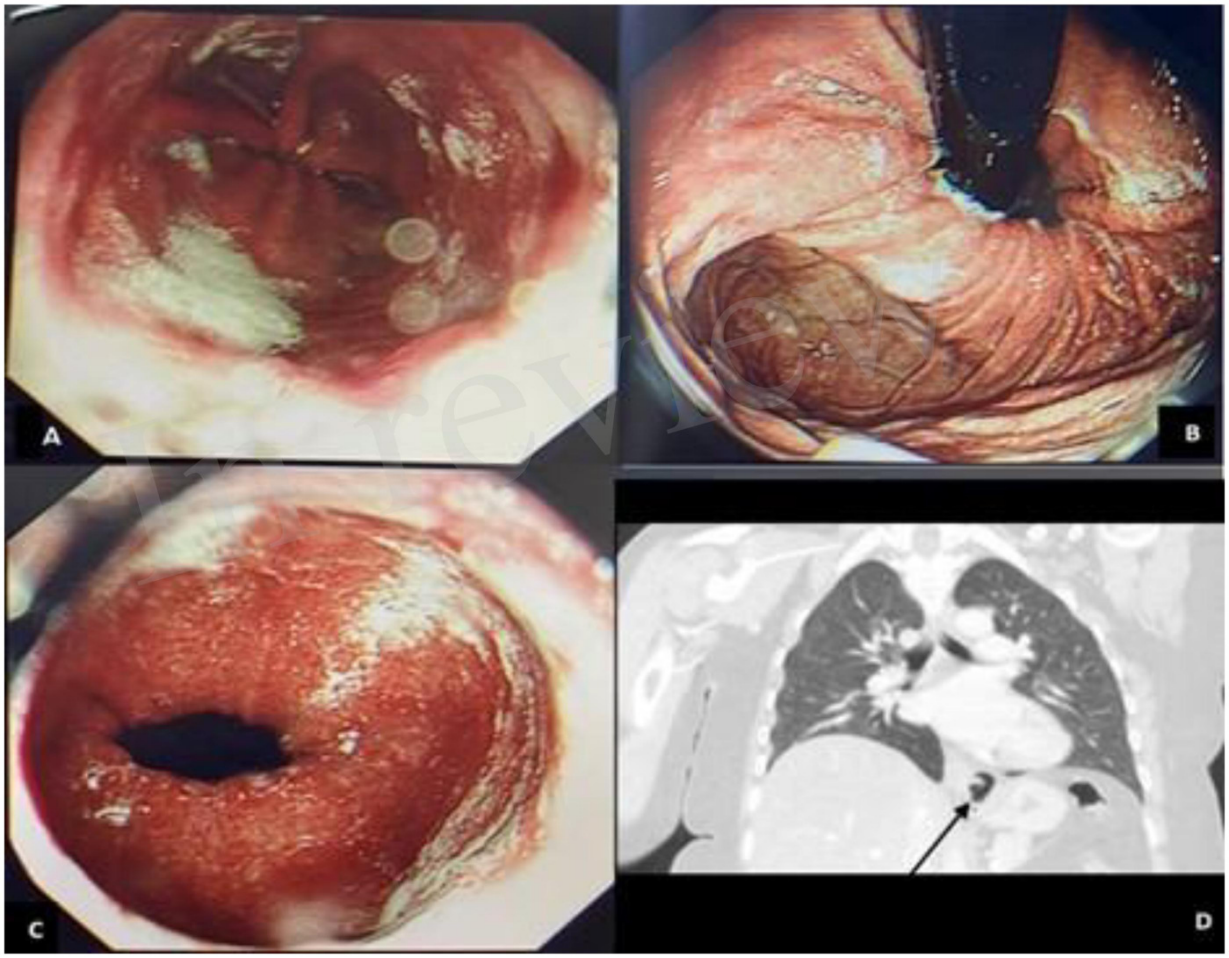
Preoperative diagnostic findings. **(A)** Erosions at the lower esophageal sphincter (LES) on endoscopy. **(B)** Endoscopic view demonstrating hiatal hernia. **(C)** Hyperemia of the prepyloric stomach. **(D)** Preoperative contrast-enhanced CT scan confirming the presence of hiatal hernia.

A 49-year-old obese woman (BMI 45 kg/m^2^), with a history of hypertension and GERD, was scheduled for laparoscopic hiatal hernia repair with Nissen fundoplication. Her medications included amlodipine 5 mg and omeprazole 40 mg daily. She had undergone three prior abdominal surgeries, including a cesarean section. Preoperative evaluation showed no respiratory or cardiovascular compromise. Diagnosis was confirmed via endoscopy (Figure 1). A barium swallow study was not performed, as the diagnosis of hiatal hernia was adequately established through endoscopy and contrast-enhanced computed tomography, in accordance with the guidelines of the Society of American Gastrointestinal and Endoscopic Surgeons (SAGES) (5).

**Figure 2:**
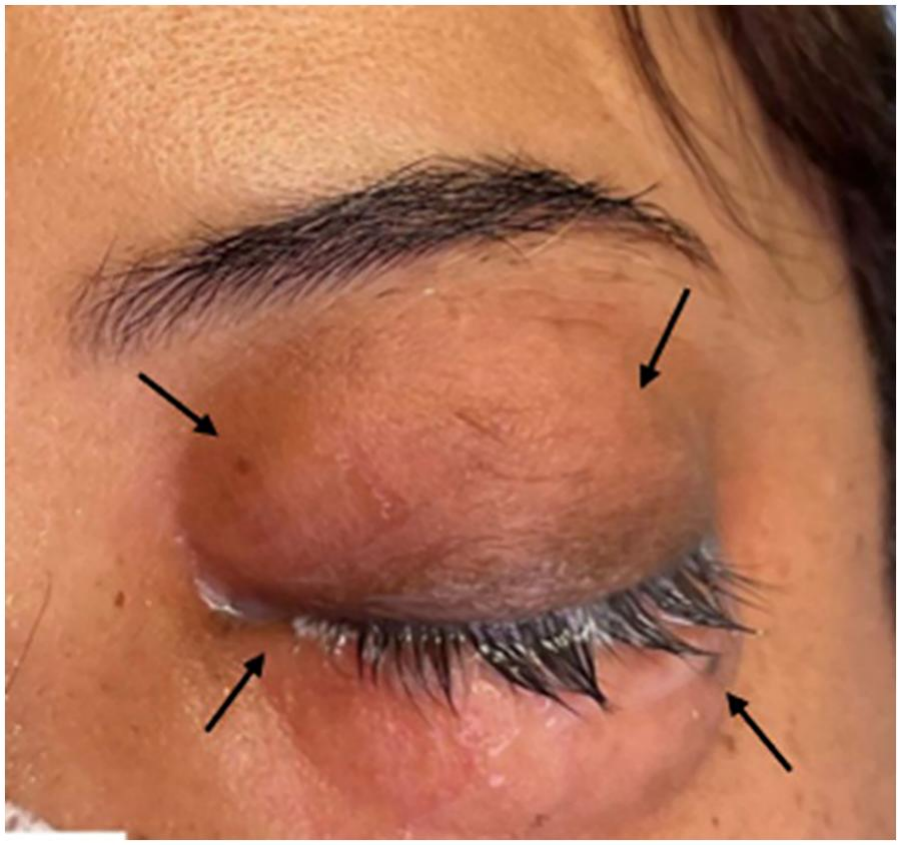
Intraoperative development of left orbital surgical emphysema. Black arrows indicate periorbital swelling and subcutaneous emphysema involving the upper and lower eyelids.

The procedure was performed under general anesthesia by an experienced team. General anesthesia was maintained using volatile anesthetic agents. Nitrous oxide was avoided during anesthesia to prevent expansion of entrapped gas. Mechanical ventilation was adjusted as needed to maintain normocapnia, with end-tidal carbon dioxide (EtCO₂) values ranging between 31 and 42 mmHg throughout the operation. EtCO_2_ remained stable within the range of 31–42 mmHg and did not demonstrate a progressive rise suggestive of significant systemic CO₂ absorption. Arterial blood gas analysis was not performed, as EtCO₂ values remained stable within acceptable limits, ventilation parameters were adequate, and there were no clinical signs of hypercarbia or respiratory acidosis. Pneumoperitoneum was established with CO₂ at 11–15 mmHg, with the patient in reverse Trendelenburg (30° head elevation). End-tidal CO₂ ranged between 31 and 42 mmHg during the 150-min operation. At port-site closure, sudden left periorbital swelling was noted (Figure 2). The abnormality was recognized by the surgical and anesthesia teams during routine visual inspection at the time of port-site closure, without preceding changes in ventilatory parameters or hemodynamic monitoring. Palpable crepitus was noted over the left periorbital region, while examination of the contralateral orbit, face, neck, and chest wall revealed no evidence of SE. The patient remained hemodynamically stable (GCS 15) with preserved visual acuity. Given facial extension, the SE was classified as Grade 3 per the Aghajanzadeh system (6).

**Figure 3:**
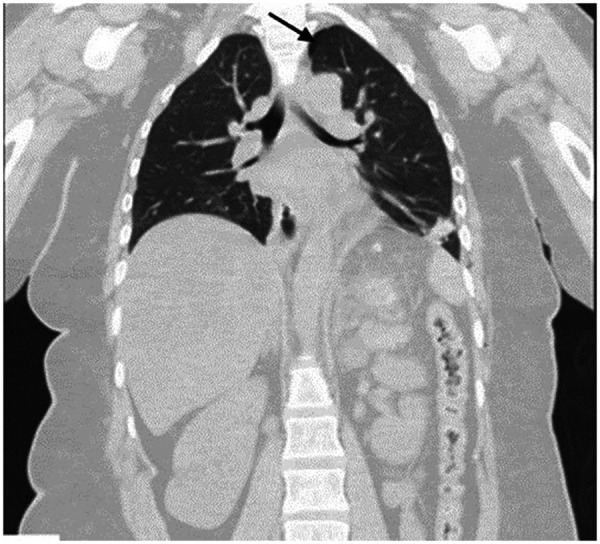
Contrast-enhanced CT chest (coronal view). Black arrow indicates mild left-sided pneumothorax at the upper lobe, with associated bilateral pleural effusions.

A chest X-ray was initially unremarkable; however, contrast-enhanced CT subsequently revealed a mild left-sided pneumothorax with bilateral pleural effusions (Figure 3). The patient was admitted to the intensive care unit (ICU) for close observation and conservative management. Within 48 h, the orbital emphysema resolved spontaneously. Given the patient's hemodynamic stability, preserved oxygenation, and absence of respiratory compromise, she was extubated uneventfully in the operating room. Admission to the intensive care unit was precautionary, in view of the extensive SE and radiologic finding of a mild pneumothorax. ICU monitoring included continuous respiratory and hemodynamic assessment, serial neurological examinations, repeat ophthalmologic evaluations, and radiologic surveillance to monitor resolution of the pneumothorax and SE. Ophthalmologic consultation was obtained promptly. Examination demonstrated best-corrected visual acuity of 20/20 in both eyes using Snellen testing, full extraocular movements without diplopia, and normal fundoscopic findings. Intraocular pressure, measured by tonometry, ranged from 12 to 16 mmHg on serial assessments. There was no proptosis, afferent pupillary defect, or evidence of orbital compartment syndrome.

The patient remained under inpatient observation for 48 h, during which complete resolution of orbital emphysema was observed. At short-term outpatient follow-up, no delayed visual, neurological, or respiratory complications were reported. A timeline of key points of patient care is outlined in Table 1.

**Table 1:** Timeline table: key events of patient care.

Time point	Event	Clinical findings/intervention	Outcome
Preoperative	Evaluation and planning	49-year-old female with GERD and prior abdominal surgeries; BMI 45 kg/m^2^; endoscopy confirmed hiatal hernia	Planned laparoscopic hiatal hernia repair with Nissen fundoplication
Intraoperative (150 min)	Laparoscopic procedure ongoing	CO₂ insufflation 11–15 mmHg, reverse Trendelenburg 30°	Stable EtCO₂ (31–42 mmHg), stable hemodynamics
Port closure	Sudden left periorbital swelling	Palpable crepitus over left orbit; no contralateral involvement; Grade 3 SE	Immediate recognition by surgical and anesthesia teams
Intraoperative imaging	CT scan and chest X-ray	Mild left pneumothorax; bilateral pleural effusions	Confirmed orbital SE; no optic nerve compression
ICU admission	Monitoring and conservative management	100% O₂ therapy, head elevation, serial ophthalmologic and neurologic checks	Hemodynamically stable; no respiratory compromise
12–24 h postop	Serial assessment	Intraocular pressure 12–16 mmHg; full ocular movements; no vision changes	Continued conservative management
48 h postop	Resolution	Orbital swelling resolved completely	Discharged with no sequelae
Follow-up	Outpatient evaluation	Preserved vision; no neurological or respiratory complications	Complete recovery

## 3 Discussion

### 3.1 Epidemiology

Laparoscopy has replaced open surgery in many fields due to its reduced recovery time and fewer adhesions. However, it introduces complications like SE due to CO₂ insufflation (7). Incidence rates vary from 0.43% to 2.34% clinically, but when using sensitive imaging, rates may reach 56% (8). Up to 77% of laparoscopic patients may develop subclinical SE, detectable only radiologically (9). Murdock et al. reported SE in 2.3% of laparoscopic cases, pneumothorax in 1.9%, and hypercarbia in 5.5%, reinforcing that CO₂-related complications are not uncommon during laparoscopy (10). Recent reports, including the study by Tamura et al., have highlighted gas-related complications during minimally invasive and robotic procedures, emphasizing the importance of early recognition and multidisciplinary management to prevent escalation to vision-threatening conditions (11).

### 3.2 Risk factors and pathophysiology

Intraoperative development of orbital emphysema is exceedingly rare. In our patient, the unilateral presentation may be attributed to asymmetry in fascial integrity or anatomical variations that permitted preferential gas tracking on the left side. Similar unilateral patterns have been described in previous case series (12). The strictly unilateral, left-sided orbital involvement observed in this case may be explained by asymmetric fascial weakness related to prior abdominal surgeries, trocar placement, and patient positioning in reverse Trendelenburg, which may have favored preferential gas tracking along left-sided anatomical planes.

Several factors predisposed this patient to orbital involvement, including morbid obesity, prior abdominal surgeries that weakened fascial planes, and the number of trocars used. Known risk factors for SE in laparoscopy include operative time >3.5 h, intra-abdominal pressure >15 mmHg, end-tidal CO₂ >50 mmHg, use of more than four trocars, and prior structural compromise (10, 13). Although the present case lasted <3 h, fascial weakness with gas tracking likely enabled orbital extension.

Morbid obesity (BMI 45 kg/m^2^) increases intra-abdominal pressure, facilitating CO₂ dissection through weakened fascial planes and likely contributing to orbital gas migration despite the absence of other high-risk features (10). CO₂ may track via the esophageal hiatus into the mediastinum, neck, and orbit when fascial resistance is reduced. The unilateral emphysema observed here suggests either asymmetric fascial defects or positional factors favoring left-sided spread (Figure 3) (4).

Risk factors for SE during laparoscopy include prolonged operative time, intra-abdominal pressures >15 mmHg, end-tidal CO₂ >50 mmHg, and use of multiple or large-caliber trocars (10, 13). Technical contributors include repeated entry attempts, improper cannula placement with stressed angulation, and inadequate fascial sealing (10). In robotic procedures, lack of tactile feedback and increased torque may further predispose to gas dissection (10). Patient-related factors such as advanced age, metabolic disease, and prior surgeries that weaken fascial planes also increase risk (10, 13). Beyond SE, CO₂ insufflation may result in pneumothorax, pneumomediastinum, hypercarbia, gas embolism, and arrhythmias, necessitating close intraoperative monitoring (7, 8, 10).

The physiologic effects of CO₂ insufflation can be significant. Severe hypercarbia may cause electroencephalographic changes (8), while gas embolism can precipitate cardiovascular collapse (10). Pneumothorax, pneumopericardium, and pneumomediastinum are additional risks, each capable of impairing ventilation or cardiac output (7, 10). Even benign findings such as periorbital swelling require vigilance due to potential visual disturbance (12). Systemic CO₂ absorption may further induce tachycardia, hypertension, and arrhythmias, emphasizing the need for close intraoperative monitoring (8, 10).

### 3.3 Clinical grading and imaging

**Figure 4:**
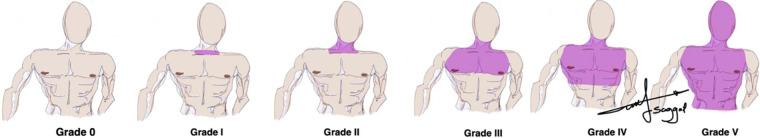
Classification of subcutaneous emphysema according to Aghajanzadeh et al. Schematic illustration showing anatomical grading: Grade 0 = trocar site only; Grade I = subcutaneous emphysema localized to the neck; Grade II = extension to the chest wall; Grade III = involvement of neck and chest wall; Grade IV = extensive extension to chest and abdominal wall; and Grade V = massive emphysema extending to face, neck, and chest.

**Figure 5:**
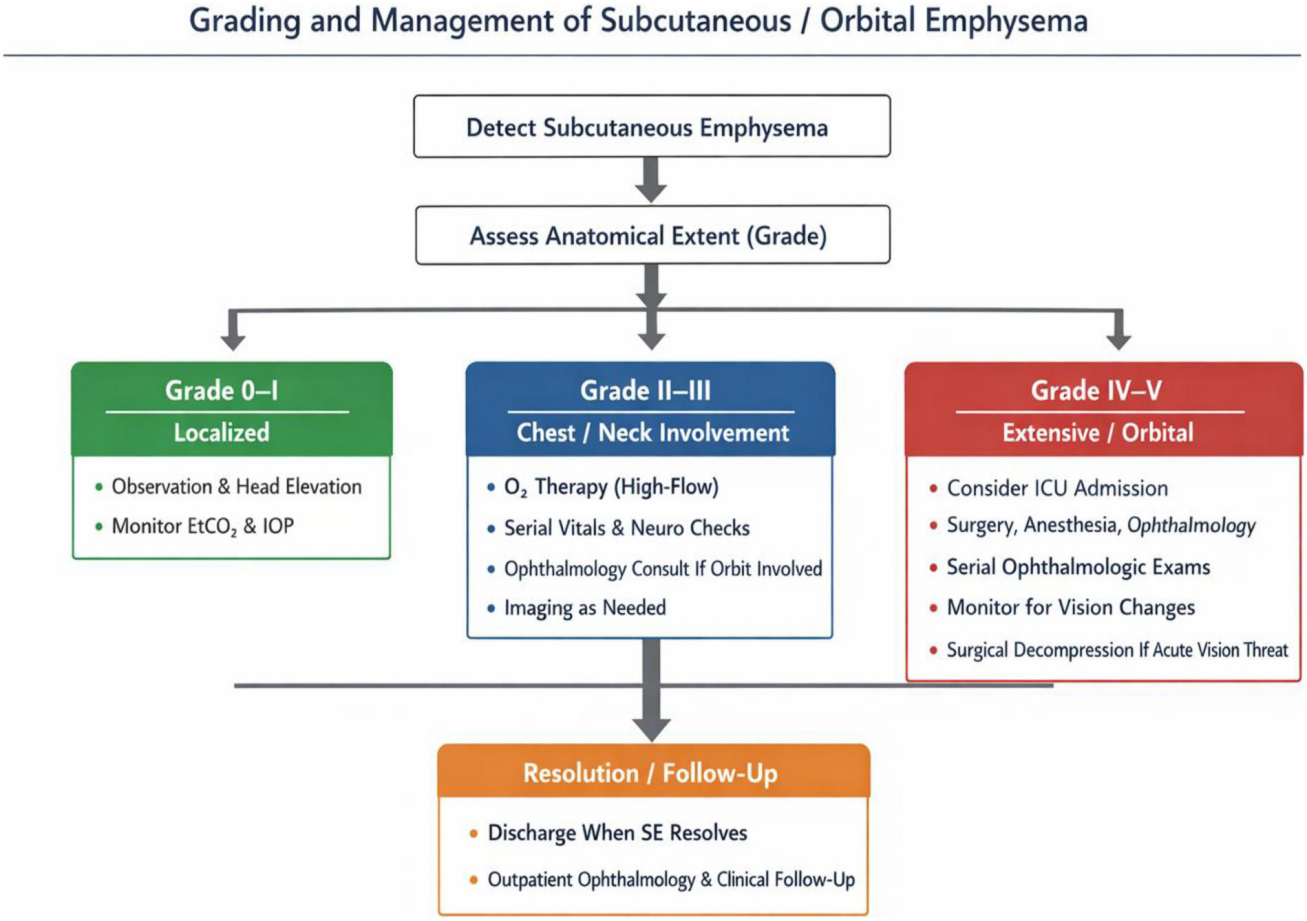
Flowchart of grading and management of subcutaneous and orbital emphysema following laparoscopic surgery.

Clinical grading of SE is essential, as lower-grade presentations typically respond to conservative measures, whereas higher-grade involvement with ocular or thoracic compromise may necessitate invasive intervention and closer monitoring. SE severity can be classified by anatomical extent, from trocar-site crepitus to massive chest and facial involvement, using a system adapted from Aghajanzadeh et al. (6). Our case met Grade 3 criteria, while orbital extension corresponds to Grade 5 in the five-grade classification (Figure 4). Figure 5 presents a schematic flowchart summarizing the grading of subcutaneous and orbital emphysema along with recommended management strategies for each grade, providing practical guidance to distinguish localized, self-limiting cases from more extensive involvement of the chest, neck, and orbit (6). Recognition relies on both clinical and physiologic indicators. Rising end-tidal CO₂, reduced lung compliance, intraoperative acidosis, and palpable crepitus serve as early warnings of severe cases requiring intervention (8).

CT is the gold standard for diagnosing orbital emphysema, clearly differentiating intraorbital air and assessing optic nerve compression (2). Chest radiographs, by contrast, may underestimate thoracic involvement, as in our patient. Orbital CT is essential in symptomatic cases with vision loss or raised intraocular pressure, but in asymptomatic patients with preserved vision and normal tonometry, conservative management avoids unnecessary radiation (14, 15). Intraoperative signs such as elevated end-tidal CO₂, reduced lung compliance, and palpable crepitus remain valuable adjuncts for early recognition (8).

### 3.4 Management

Most orbital SE cases are self-limiting and respond well to conservative measures, including high-flow oxygen to promote reabsorption of subcutaneous air through nitrogen washout (1), bed rest with head elevation, and serial monitoring of intraocular pressure when the orbit is involved (2).

Surgical intervention, such as needle decompression, is rarely required but may be considered if vision is acutely threatened or intraocular pressure rises significantly (6). In our patient, intraocular pressure remained within normal limits during 12-h assessments, and symptoms resolved without invasive intervention.

### 3.5 Prevention

Preventive strategies are essential in laparoscopy, particularly for high-risk patients. Core measures include maintaining insufflation pressures <15 mmHg, ensuring tight fascial seals, minimizing trocar use, reducing operative time, and closely monitoring end-tidal CO₂ (10, 13). Intraoperative warning signs, such as facial swelling, crepitus, or hypercarbia, should prompt suspicion of SE, with prompt recognition and imaging guiding management. Once SE is identified, recommended steps include lowering intra-abdominal pressure, discontinuing nitrous oxide, delivering 100% oxygen, and increasing minute ventilation to enhance CO₂ clearance (10). Patients should also be assessed for pneumothorax before extubation to ensure airway safety (10). Ultimately, meticulous technique, pressure control, careful trocar handling, and vigilant monitoring of CO₂ remain central to prevention (13).

While orbital emphysema is often self-limiting, it is crucial to differentiate early-onset (intraoperative) from delayed onset (postoperative) cases. Early onset can suggest rapid gas migration, often associated with fascial defects or improper trocar sealing. Early recognition is vital to prevent escalation into orbital compartment syndrome. Favorable outcomes in such cases depend not only on early recognition but also on appropriate anesthetic management, multidisciplinary coordination, and vigilant perioperative monitoring.

## 4 Patient perspective

The patient reported experiencing anxiety related to the sudden facial swelling but expressed reassurance after being informed that her vision was unaffected. She was satisfied with the conservative management approach and relieved by the rapid resolution of symptoms.

## Data Availability

The original contributions presented in the study are included in the article/Supplementary Material, further inquiries can be directed to the corresponding author.
